# Deep learning-based applicator selection between Syed and T&O in high-dose-rate brachytherapy for locally advanced cervical cancer: a retrospective study

**DOI:** 10.1088/1361-6560/addea5

**Published:** 2025-06-06

**Authors:** Runyu Jiang, Malvern Madondo, Xiaoman Zhang, Yuan Shao, Mohammadamin Moradi, James J Sohn, Tianming Wu, Xiaofeng Yang, Yasmin Hasan, Zhen Tian

**Affiliations:** 1 Department of Radiation & Cellular Oncology, University of Chicago, Chicago, IL, United States of America; 2 Department of Physics, University of Chicago, Chicago, IL, United States of America; 3 School of Public Health, University of Illinois Chicago, Chicago, IL, United States of America; 4 Department of Radiation Oncology, Emory University, Atlanta, GA, United States of America

**Keywords:** deep learning, high-dose-rate brachytherapy, locally advanced cervical cancer, applicator selection, decision-making

## Abstract

*Objective.* High-dose-rate (HDR) brachytherapy is integral to the standard-of-care for locally advanced cervical cancer (LACC). Currently, selection of brachytherapy applicators relies on physician’s clinical experience, which can lead to variability in treatment quality and outcomes. This study presents a deep learning-based decision-support tool for selecting between interstitial Syed applicators and intracavitary tandem & ovoids applicators. *Approach.* The network architecture consists of six 3D convolutional-pooling-rectified linear unit blocks, followed by a fully connected block. The input to the network includes three channels: a 3D contour mask of clinical target volume (CTV), organs at risk (OAR), and central tandem, and two 3D distance maps of CTV and OAR voxels relative to the tandem’s central axis. The network outputs a probability score, indicating the suitability of Syed applicators. Binary cross-entropy loss combined with *L*_1_ regularization was used for network training. *Main results.* A retrospective study was performed on 184 LACC patients with 422 instances of applicator insertion. The data was divided into three sets: Dataset-1 of 163 patients with 372 insertions for training and hyperparameter tuning, Dataset-2 of 17 patients with 36 insertions and Dataset-3 of four complex cases with 14 insertions for testing. Five-fold cross-validation was performed on Dataset-1, during which hyperparameters were heuristically tuned to optimize classification accuracy across the folds. The highest average accuracy was 92.1 ± 3.8%. Using the hyperparameters that resulted in this highest accuracy, the final model was then trained on the full Dataset-1, and evaluated on the other two independent datasets, achieving 96.0% accuracy, 90.9% sensitivity, and 97.4% specificity. *Significance.* These results demonstrate the potential of our model as a quality assurance tool in LACC HDR brachytherapy, providing feedback on physicians’ applicator choice and supporting continuous improvement in decision-making. Future work will focus on collecting more data for further validation and extending its application for prospective applicator selection.

## 1. Introduction

Cervical cancer is one of the leading causes of cancer-related death among women worldwide (Torre *et al* 2017, Bray *et al* 2018). For patients with locally advanced cervical cancer (LACC), the standard treatment approach typically involves pelvic external beam radiotherapy (EBRT) combined with concurrent chemotherapy, followed by high-dose-rate (HDR) brachytherapy (Nag *et al* 2000b, Viswanathan *et al* 2012, Han *et al* 2013, Pinn-Bingham *et al* 2013, Banerjee and Kamrava 2014, Le Guyader *et al* 2022). In HDR brachytherapy, applicators are inserted either intracavitary or interstitially, positioning the radioactive source Ir-192 within or near the tumor (Nag *et al* 2000a). Due to its steep dose fall-off, HDR brachytherapy allows for dose escalation in the high-risk clinical target volume (HR-CTV) while sparing surrounding healthy tissues and organs at risk (OARs), thus enhancing local control and improving survival rates (Georg *et al* 2008, Karlsson *et al* 2017).

The effectiveness of HDR brachytherapy largely depends on the selection of an appropriate applicator, which determines the radioactive source’s path within the body and the resultant dose distribution (Sawicki 2017, Kallis *et al* 2021, Sohn *et al* 2022). The GEC-ESTRO group highlights the importance of choosing the appropriate applicator to achieve optimal dose distribution to the HR-CTV while minimizing exposure to normal tissues (Fokdal *et al* 2016, Fortin *et al* 2016). While clinical guidelines provide general recommendations for applicator selection based on HR-CTV volume and lateral extent (Haie-Meder *et al* 2005, Pötter *et al* 2006, Viswanathan *et al* 2012), the spatial relationship between the CTV and adjacent OARs is also crucial for choosing an applicator that maximizes OAR sparing. Currently, applicator selection often relies on a physician’s experience and judgement, which can lead to inconsistencies in selections and suboptimal treatment plans (Kallis *et al* 2021, Stenhouse *et al* 2021). This concern is particularly pronounced for less experienced physicians. Therefore, there is a pressing need for a patient-specific, objective approach to applicator selection to ensure consistent and optimal treatment quality.

To address this need, Yoshida *et al* conducted a simulation study comparing three types of applicators by modeling various HR-CTV sizes and generating treatment plans for each applicator type (Yoshida *et al* 2016). The study included intracavitary tandem & ovoids, interstitial Syed applicators, and a hybrid intracavitary/interstitial approach combining tandem & ovoids with a few interstitial needles. Their simulation results suggested that intracavitary tandem & ovoids applicators are preferable for HR-CTV volumes smaller than 4 × 3 × 3 cm^3^. However, the simplified rectangular models used for the HR-CTV, bladder and rectum, along with fixed spatial distances between structures, limits the applicability of this finding to real-world clinical scenarios with more complex patient anatomies. More recently, Stenhouse *et al* developed a machine learning (ML)-based approach to aid in selecting between traditional intracavitary options (i.e. tandem & ovoids, tandem & ring) and a hybrid intracavitary/interstitial approach (i.e. tandem & ring with a few interstitial needles), achieving an accuracy of 91.5% (2021). While this ML approach could potentially be adapted to other applicator types, its reliance on hand-crafted features imposes significant limitations on its flexibility. Adapting the model to new applicators would likely require users to manually extract relevant features specific to those applicators. Without the proper features manually extracted by the users, even with advanced feature selection methods, the ML model is unlikely to achieve optimal performance.

Compared to traditional ML methods, deep learning (DL) can learn hierarchical representations and complex patterns without the need for manual feature extraction, making it well-suited for recognizing intricate anatomical variations. With numerous successful DL applications in healthcare, particularly in EBRT (Wang *et al* 2020, Boulanger *et al* 2021, Lin *et al* 2021, Samarasinghe *et al* 2021, Appelt *et al* 2022, Lastrucci *et al* 2024), recent efforts have explored DL for various tasks in cervical HDR brachytherapy, including automatic detection and digitization of applicators (Jung *et al* 2019, Xie *et al* 2023, Wang *et al* 2024), OAR delineation (Mohammadi *et al* 2021, Li *et al* 2022, Zhu *et al* 2024), dose prediction (Ma *et al* 2021, Kallis *et al* 2023, Li *et al* 2023), and automatic treatment planning (Shen *et al* 2019, Pu *et al* 2022, Kallis *et al* 2023). However, the potential of DL for applicator selection remains unexplored. In this study, we aim to harness the capabilities of DL to develop a robust, patient-specific decision-making tool for applicator selection in LACC HDR brachytherapy. At our institution, the intracavitary tandem & ovoids applicators (referred to as T&O hereafter) and the interstitial Syed applicators using a perineal template that allows the insertion of an intracavitary tandem and multiple interstitial needles (referred to as Syed hereafter) are the two most used applicator types for LACC HDR brachytherapy. Compared to T&O, Syed applicators offer more flexible and customized dose control to improve target coverage for tumors with large, irregular or asymmetric shapes while sparing surrounding OARs. However, this dosimetric advantage comes with increased procedural invasiveness, complexity, and resource demands, as well as patient discomfort and greater risks of infection and injury to surrounding organs. In this study, we developed a deep neural network to predict the suitability of Syed versus T&O applicators for individual LACC patients based on their specific anatomy. Our study not only aims to assist in selecting the optimal applicator type between these two options to achieve acceptable plan quality, but also seeks to minimize the unnecessary use of invasive Syed applicators, ultimately improving overall patient care in LACC HDR brachytherapy.

## 2. Materials and methods

### 2.1. Patient data

With approval from our institutional review board (IRB), we retrospectively collected data from 184 LACC patients who received HDR brachytherapy with either Syed or T&O applicators at our institution since the launch of our HDR program in 2013. Most Syed treatments involved a single applicator insertion procedure, followed by three to five treatment fractions, while a small subset underwent two separate insertions, each followed by two to three fractions, due to suboptimal needle placement in the first insertion or other clinical considerations. In contrast, patients treated with T&O applicators were generally managed as outpatients, with separate applicator insertion for each treatment fraction, while a smaller subset admitted as inpatients underwent a single insertion for multiple fractions. For each patient, we collected 3D CT images acquired post-insertion, along with contours of the HR-CTV, intermediate-risk clinical target volume (IR-CTV) when applicable, and OARs including the bladder, small bowel, large bowel, rectum, and sigmoid. These contours were manually delineated by physicians on CT images, complemented by post-insertion MRI images, for treatment planning. We also collected the coordinates of the central tandem that was digitalized by medical physicists during treatment planning, as well as the resulting treatment plans. It is important to note that the applicators in these collected cases were selected based solely on the physician’s experience and judgement, which might not reflect the true optimal choice in atypical cases. To establish a ground truth for the optimal applicator type, an experienced medical physicist specializing in cervical HDR brachytherapy reviewed all clinical treatment plans. Specifically, for T&O cases, if the dosimetric quality metrics (e.g. *D*_90%_ for HR-CTV and IR-CTV, *D*_2cc_ for OARs) met our planning guidelines, the T&O applicator was confirmed as the ground truth; otherwise, the ground truth type was relabeled as Syed, based on the assumption that Syed provides greater dosimetric flexibility. For Syed cases, the physicist simulated a T&O insertion and created a T&O plan for dosimetric quality comparison. If the simulated T&O plan quality was comparable to the Syed plan, the ground truth applicator label was assigned as T&O; otherwise, Syed was retained as the ground truth applicator. The verified ground true applicator type was then used in our study.

We divided the collected cases into three independent subgroups. (1) Dataset-1: Serving as the training data, it includes data from 163 patients treated between 2013 and September 2023. Of these patients, 95 were treated with Syed applicators and 68 with T&O applicators, totaling 372 applicator insertion procedures (i.e. 100 Syed insertions and 272 T&O insertions); (2) Dataset-2: Used as one of the testing datasets, this set comprises 17 patients treated after September 2023, of whom 7 were treated with Syed applicators and 10 with T&O. This set contains 36 insertion procedures in total, including 7 Syed and 29 T&O insertions; (3) Dataset-3: Used as an additional testing dataset, this set includes four patients treated at our institution, whose applicator type was switched during the HDR brachytherapy course due to factors such as rapid tumor regression, progression, or initial applicator mis-selection. The detailed information for these four cases is provided below:

• Case 1: The first two insertions used T&O applicators. An MRI scan was acquired between the second and the third insertions and revealed significant tumor progression, prompting the use of a Syed applicator in the third insertion.
• Case 2: This patient had three insertions, with the first two using a T&O applicator. For the third insertion, significant tumor progression (HR-CTV volume increased from 35.8–35.9 cc to 77.1 cc) rendered the T&O applicator, which was used in the actual insertion, insufficient, leading to clinically unacceptable plan quality. As a result, the treatment delivery of this T&O plan was canceled, and the patient was rescheduled for a fourth insertion with a Syed applicator.
• Case 3: This patient had five insertions, with the first four using T&O applicators and the fifth using a Syed applicator. Since the treatment took place in 2015 and the HR-CTV volume for the fifth insertion was similar to that of the previous insertions, the reason for switching to a Syed applicator is unclear and difficult to recall due to the time lapse.
• Case 4: In this case, the first insertion used a Syed applicator, while the second insertion, performed two weeks later, used a T&O applicator due to tumor regression.

These cases in Dataset-3 provide a valuable test for evaluating the efficacy and robustness of our method in managing such complex clinical scenarios.

### 2.2. Network architecture

**Figure 1.**
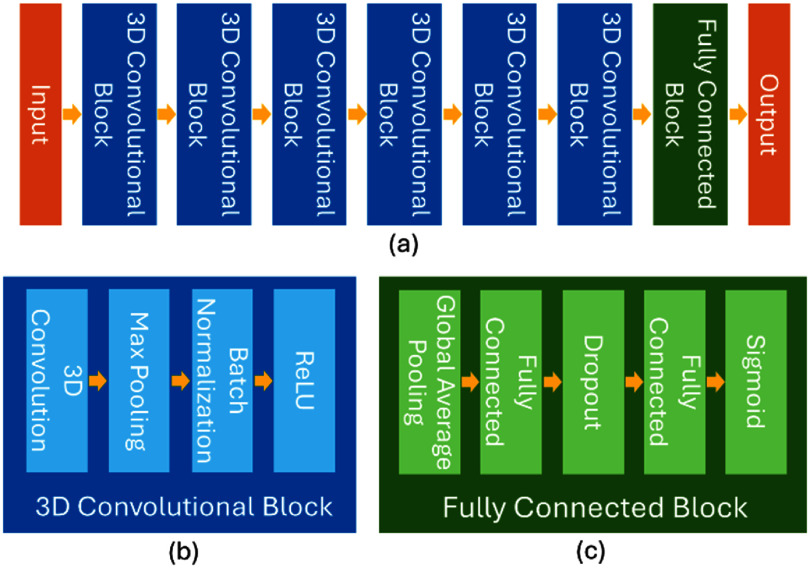
Network architecture of our DL-based decision-making tool for applicator selection. The subfigure (a) illustrates the overall architecture, and the subfigures (b) and (c) present the detailed architecture for the 3D convolutional block and the fully connected block, respectively.

We frame the applicator selection problem as a classification task, and develop a deep neural network to extract and learn relevant features from a patient’s 3D anatomy to predict the suitability of Syed applicators. As illustrated in figure 1, our network consists of six 3D convolutional blocks followed by a fully connected block. Each convolutional block contains a 3D convolutional layer, a max pooling layer, a batch normalization layer, and a rectified linear unit activation layer. The fully connected block comprises a global average pooling layer, two fully connected layers with a dropout layer in between, and a Sigmoid activation function to ensure the output ranges between 0 and 1. This network architecture was empirically designed. The specifications of the six 3D convolutional blocks and the two fully connected layers in the fully connected block are detailed in table 1. The whole network contains a total of 3409 trainable parameters. In our study, we used the default threshold of 0.5 for classification, meaning that an output of 0.5 or greater indicates that the Syed applicator type is recommended, while an output below 0.5 suggests that a T&O applicator is sufficient.

**Table 1.** Specifications of network architecture.

		Zero padding size	No. of filters	Filter size	Max pooling size
3D convolutional blocks	Block 1	(1, 1, 1)	4	(3, 2, 2)	(3, 3, 3)
	Block 2	(1, 1, 1)	4	(2, 2, 2)	(2, 2, 2)
	Block 3	(1, 1, 1)	8	(3, 3, 3)	(2, 2, 2)
	Block 4	(2, 1, 1)	8	(3, 2, 2)	(2, 2, 2)
	Block 5	(1, 1, 1)	4	(3, 3, 3)	(2, 2, 2)
	Block 6	(1, 1, 1)	4	(3, 3, 3)	(2, 2, 2)

Fully connected block	Layer 1	N/A	8 neurons	N/A	N/A
	Layer 2	N/A	1 neuron	N/A	N/A

### 2.3. Input data preparation

Given that TG-43 dose calculation is used in our clinical practice of cervical HDR brachytherapy, which assumes homogenous tissue density, we excluded image density of patient’s 3D CT images and instead focused on the contours of the CTVs and OARs as the primary inputs for our analysis. To well represent factors relevant to applicator selection, such as the volume size, symmetry, and lateral extent of the CTVs, and their spatial relationships to nearby OARs, we preprocessed the contour points derived from the DICOM-RT structure files.

**Figure 2.**
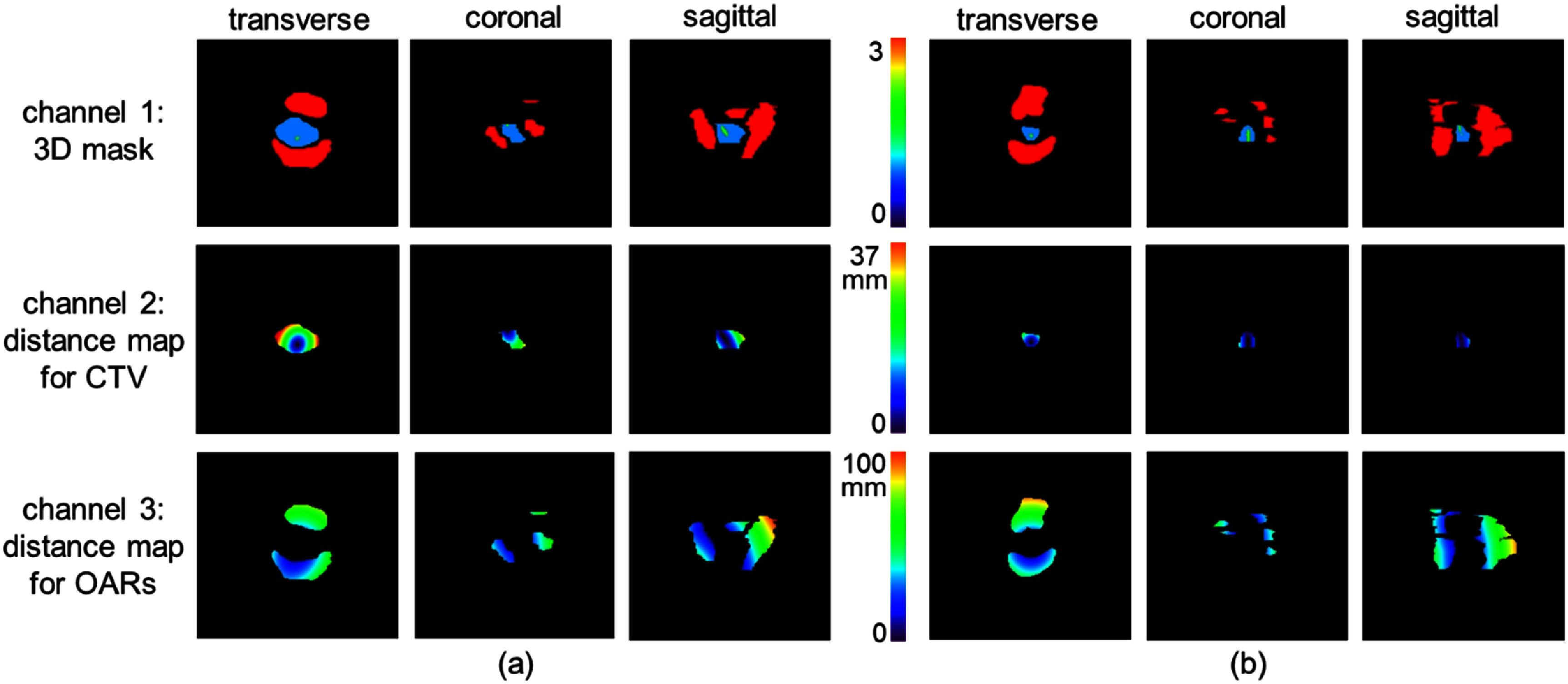
Illustrations of the network’s 3-channel input data for a Syed case (a) and a T&O case (b) in the views of transverse, coronal and sagittal. Each row corresponds to a channel of the input data.

Specifically, we generated a 3D structure mask aligned with the CT image coordinates. The symmetry of the target volume relative to the cervical canal often informs the applicator selection for LACC HDR brachytherapy. However, the cervical canal is not contoured in our clinical practice during treatment planning. Therefore, in this retrospective study, we used the central tandem, which is inserted through the vaginal canal, external os, cervical canal, internal os, and into the uterine cavity for both Syed and T&O applicators, as an indicator for the cervical canal’s location. This central tandem, digitized by medical physicists during treatment planning and stored in the DICOM-RT structure file, was represented in our mask data and expanded by 5 mm in diameter to enhance its representation and facilitate display. In the 3D mask, a value of 1 indicates voxels within the CTV volume. For patients with only the HR-CTV as the treatment target, the CTV mask was generated for the HR-CTV alone. For cases where the IR-CTV was also included, the generated CTV mask combined the HR-CTV and IR-CTV volumes. A value of 2 denotes voxels representing the central tandem. A value of 3 marks voxels within any OARs involved in LACC HDR brachytherapy (e.g. bladder, rectum, sigmoid, small bowel, large bowel). This 3D mask data formed the first input channel for our network. While the mask data provides relative spatial relationships between the CTV and OARs, it does not convey actual physical size or distance information, which are also essential for applicator selection. To address this, we generated two 3D distance maps as the second and third input channels. The first distance map represents the distance, in millimeters, for each voxel in the CTV relative to the central tandem, while the second map represents the distance for each voxel in the OARs relative to the central tandem. The processed 3D mask data and the two distance maps were resampled to a uniform resolution of 2 × 2 × 2 mm^3^ and truncated to a dimension of 165 × 176 × 176. To preserve the physical distance information, no initial normalization was applied to the data. Figure 2 illustrates the preprocessed 3-channel input data for a Syed case and a T&O case.

### 2.4. Network training

The network was developed and trained using TensorFlow. The binary cross-entropy loss was employed to supervise network training. Given the limited availability of patient data, an *L*_1_ regularization term was incorporated into the loss function to enforce sparsity in the network weights to help mitigate overfitting. The total loss function is defined as:

\begin{equation*}L = - \frac{1}{N}\mathop \sum \limits_{i = 1}^N \left( {{y_i}\log \left( {{p_i}} \right) + \left( {1 - {y_i}} \right){\text{log}}\left( {1 - {p_i}} \right)} \right) + \lambda \mathop \sum \limits_{j = 1}^M \left| {{W_j}} \right|.\end{equation*}

The first term is the binary cross-entropy, where $N\,$ represents the batch size, and ${y_i}$ denotes the true applicator label for the $i{\text{th}}$ sample in the batch, with a value of 1 for Syed and 0 for T&O. The network output, ${p_i}$, represents the predicted suitability score for using Syed for the $i{\text{th}}$ sample. The second term represents the *L*_1_ regularization applied to the network parameters, with $M\,$ as the total number of trainable network parameters, ${W_j}$ as the $j{\text{th}}$ parameter, and $\lambda \,$ as the relative weight of the regularization.

To further reduce overfitting, we applied data augmentation to increase the diversity of the training data. Our augmentation strategies included translations in three directions (i.e. superior-inferior, left-right, anterior-posterior), rotations of up to 10° around the superior-inferior axis, and left-right flips. Each training sample had a 75% probability of undergoing augmentation, with equal chances of being translated or rotated. Following translation or rotation, each sample then had a 50% probability of undergoing a left-right flip. These augmentations simulate realistic setup variations during CT image acquisition, while the restriction of rotations to the superior-inferior axis and flips to the left-right direction aligns with the supine positioning and head-first scanning protocol for LACC patients in HDR brachytherapy and accounts for the anatomical relationships of the cervix and uterus within the patient’s body. Additionally, no deformations were applied during augmentation, as maintaining accurate volume size and spatial relationships is crucial for applicator selection.

To optimize network performance, we performed five-fold cross-validation for hyperparameter tuning, adjusting hyperparameters such as the relative weight of *L*_1_ regularization, learning rate, and dropout rate. Specifically, the training dataset, Dataset-1, was divided into five groups, with four groups used for training and the remaining one for validation in each fold. Since input data from different insertions of the same patient are likely to share high similarity, data splitting was performed at the patient level, ensuring that all insertions from a single patient were placed in the same group. This approach prevents the network from accessing information from the same patient during both training and validation, thus avoiding potential bias. To address the class imbalance between Syed and T&O cases, weighted sampling was employed during training. Specifically, each batch was filled with randomly selected patient cases from the training data. The selection process ensured balanced sampling, with each sampling in the batch having a 50% probability drawn from Syed patients in the training set and 50% probability drawn from T&O patients. In cases where a patient had multiple insertions, one insertion was randomly chosen for inclusion in the batch. This approach ensured that the network learned from each applicator type with equal frequency. For each set of hyperparameters, the average classification accuracy across all five folds was calculated, and the hyperparameters were adjusted heuristically to improve this average accuracy. After identifying the optimal hyperparameter values, the network was retrained on the full training dataset and evaluated on two testing datasets, Dataset-2 and Dataset-3, which were unseen during training.

### 2.5. Model performance evaluation

#### 2.5.1. Evaluation metrics

To evaluate our model’s performance in our retrospective study, we used classification accuracy as a primary metric for assessing its ability in selecting the appropriate applicator type. Since one of our clinical goals is to avoid the unnecessary use of the invasive Syed applicators while ensuring acceptable plan quality, we further employed sensitivity and specificity as evaluation metrics. By defining cases requiring a Syed applicator as the ‘positive’ cases, sensitivity measures the model’s ability to correctly identify cases where the invasive applicator is necessary for a clinically acceptable plan, while specificity evaluates the model’s effectiveness in avoiding invasive procedures when an intracavitary T&O is sufficient to enable acceptable plans.

#### 2.5.2. Benchmark with conventional ML -based approach

For benchmarking purposes, we employed four conventional ML methods, including support vector machine (SVM), random forest (RF), K-nearest neighbors (KNN), and decision tree (DT). Their performance was compared with that of our proposed DL model.

Based on our clinical experience, we extracted 20 handcrafted features to characterize the geometric properties of the CTV volume and its spatial proximity to OAR. The CTV geometric features include:

• The size of HR-CTV, or the combined volume of HR-CTV and IR-CTV when both were present;
• The average and maximum lateral extension of the CTV relative to the central tandem, calculated across all CTV-involved axial image slices;
• The average and maximum offset between the center of mass of the CTV (per image slice) and the central tandem, serving as a measure of CTV asymmetry.

Given the clinical relevance of the *D*_2cc_ dose to OARs in LACC HDR brachytherapy, we also quantified the spatial proximity of each OAR (e.g. bladder, rectum, sigmoid, small bowel, large bowel) to the CTV. Specifically, for each OAR, we calculated the distance from each voxel to the CTV surface, identified the nearest 2cc OAR volume, and then computed the maximum, minimum, and average distance among these voxels to the CTV surface.

All ML experiments were conducted using the Scikit-Learn package in Python (Pedregosa *et al* 2011). To assess the relevance and predictive value of each handcrafted feature, we performed an ANOVA *F*-test and ranked features by their *F*-scores. Features with higher *F*-scores were considered more informative for applicator type selection. Similar to our DL experiments, hyperparameter tuning was performed for each ML model using grid search with five-fold cross-validation on Dataset-1. Since the optimal number of features was unknown, we repeated this process using varying numbers of top-ranked features. To address the class imbalance between Syed and T&O cases in Dataset-1, we employed the synthetic minority oversampling technique, which generates synthetic samples for the minority class (i.e. Syed cases) using a k-nearest neighbor approach during model training (Chawla *et al* 2002). Once the optimal number of features and hyperparameters of each model were determined, the four ML models were retrained using the complete Dataset-1. Finally, the predictions from the four final models were aggregated using a soft-voting ensemble to generate the prediction results for Dataset-2 and Dataset-3.

## 3. Results

### 3.1. Five-fold cross-validation and final model training on dataset-1

**Figure 3.**
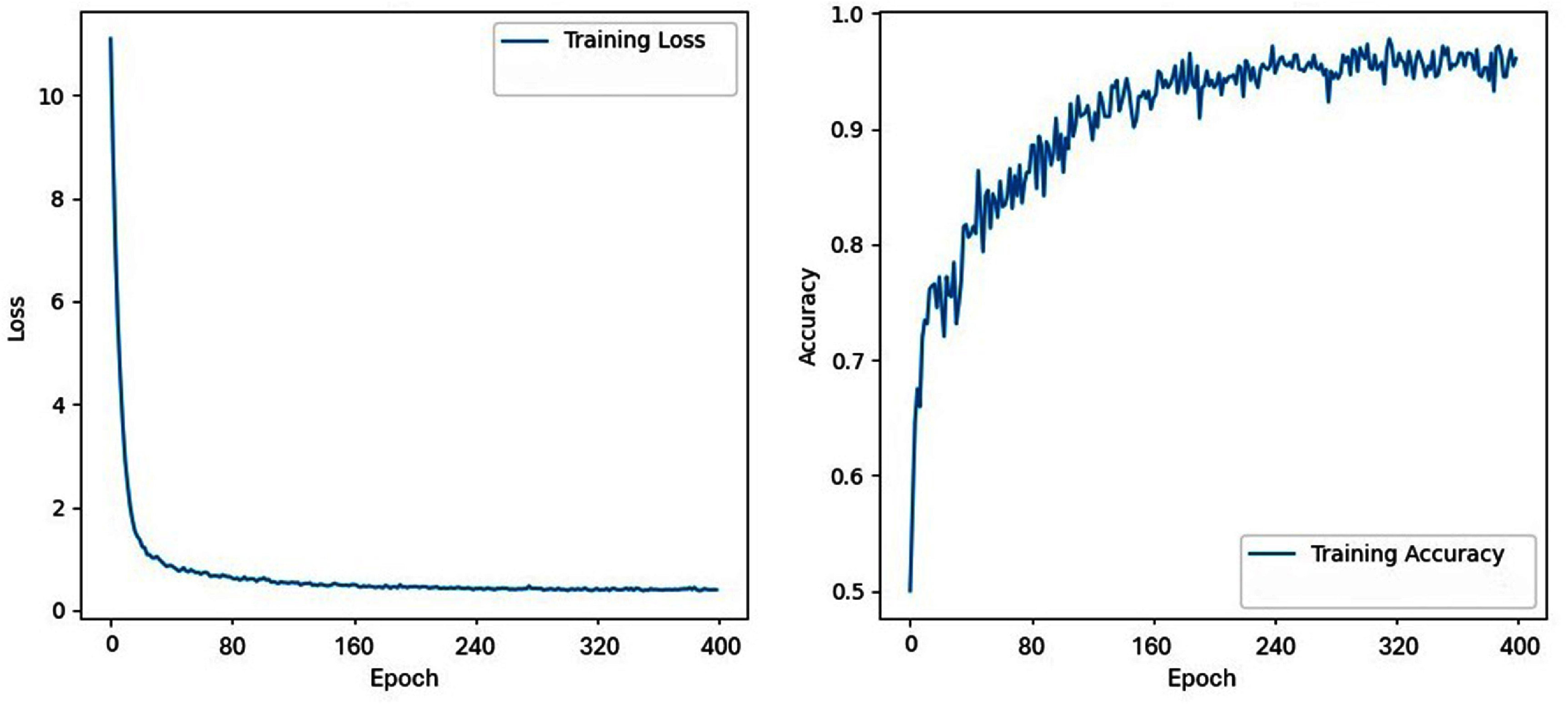
Training curves of loss (left) and accuracy (right) during the final training process of our DL model.

During five-fold cross-validation, the training process of each fold ran for 400 epochs, with each epoch comprising 40 batches of 8 samples per batch. The training samples within the randomly selected four groups of Dataset-1 were processed approximately once per epoch. Through heuristic hyperparameter tuning over many rounds of cross-validation, the highest average validation accuracy across all the five folds was 92.1 ± 3.8%. Here, the validation accuracy is defined per insertion. Detailed five-fold cross-validation results are provided in table 2. The hyperparameter values that yielded the highest accuracy were selected for final model training, which include an *L*_1_ regularization weight of 0.03, an exponentially decaying learning rate starting at 0.001 and decaying by a factor of 0.93 every 200 batches, and a dropout rate of 10%. The final model was trained on the complete Dataset-1 over 400 epochs, with each epoch comprising 50 batches of 8 samples, covering the entire dataset once per epoch. Figure 3 shows the training loss and training accuracy over the course of this training process.

**Table 2.** Five-fold cross validation results of our proposed DL method obtained with identified optimal hyperparameter values.

Fold	Training loss	Training accuracy (%)	Validation loss	Validation accuracy (%)
1	0.392	96.9	0.369	98.6
2	0.384	95.3	0.436	91.6
3	0.373	97.7	0.502	91.4
4	0.410	97.2	0.534	90.4
5	0.400	98.4	0.636	88.5

Mean ± std	0.392 ± 0.013	97.1 ± 1.1	0.495 ± 0.095	92.1 ± 3.8

### 3.2. Model evaluation on Dataset-2 and Dataset-3

The final model was first tested on Dataset-2, none of which were seen by the network during training. As shown in table 3, all 7 Syed insertions were correctly classified, with predicted Syed suitability scores ranging from 0.585 to 0.771. Among the 29 T&O insertions, 28 were correctly classified, with Syed suitability scores ranging from 0.003 to 0.323. One T&O insertion was misclassified, with a predicted Syed suitability score of 0.542. Considering Syed insertions as positive cases (indicating the need for invasive Syed applicators), our final model achieved 100.0% sensitivity, 96.6% specificity, and 97.2% accuracy.

**Table 3.** Overall model performance of the final DL model on Dataset-2 and Dataset-3.

	No. of positive samples	No. of negative samples	No. of true positive	No. of false positive	No. of true negative	No. of false negative	Sensitivity (%)	Specificity (%)	Accuracy (%)
Dataset-2	7	29	7	1	28	0	100.0	96.6	97.2
Dataset-3	4	10	3	0	10	1	75.0	100.0	92.9

Total	11	39	10	1	38	1	90.9	97.4	96.0

**Figure 4.**
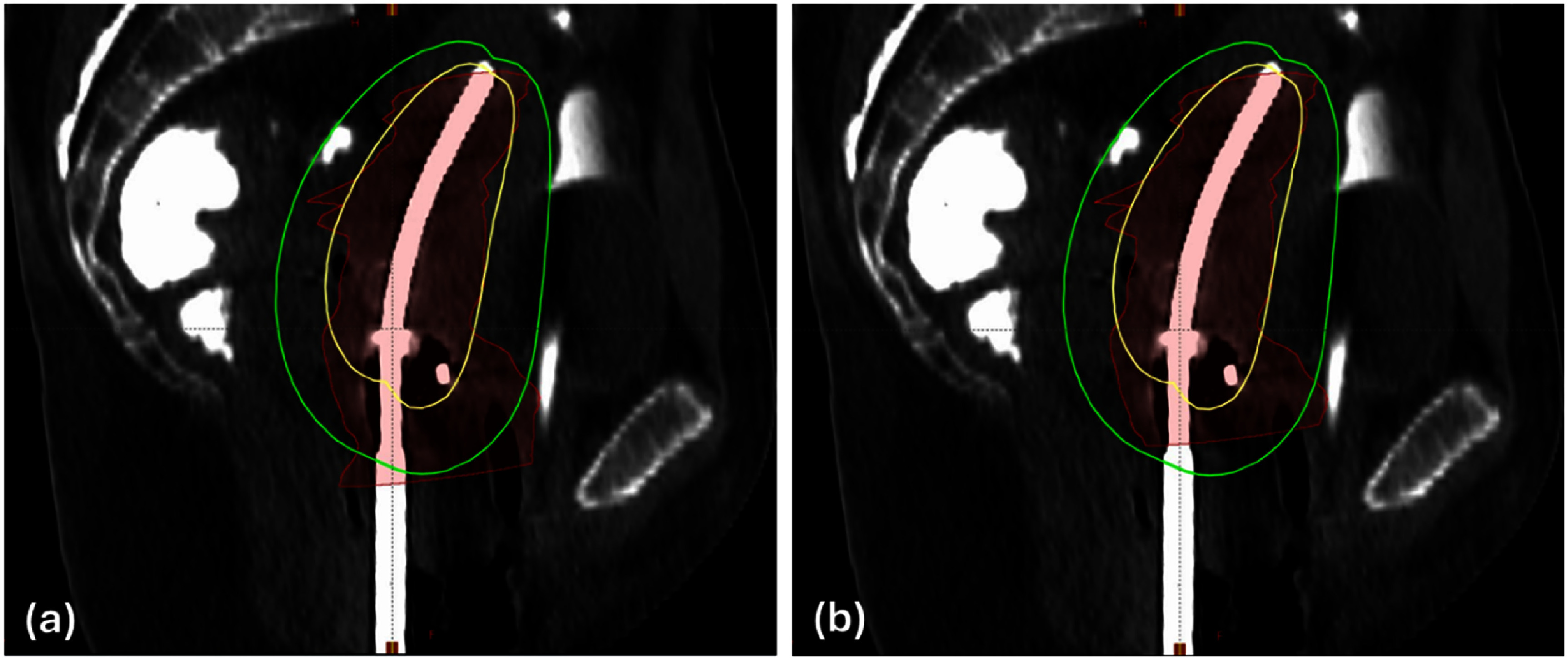
Illustration of HR-CTV contour (red) for the first insertion of the case 3 with a T&O applicator inserted. The isodose lines of 100% and 50% of the prescription dose from the clinical treatment plan are shown in yellow and green, respectively. Subfigure (a) displays the original HR-CTV contour, which deviates from conventional contouring practices that most T&O cases follow, with a substantial inferior portion not covered by the prescription dose and even a significant area not covered by the 50% prescription dose. Subfigure (b) shows our modified HR-CTV contour, where the contours on the last five inferior CT slices were removed. The results of our final model, using both the original and modified contours, are presented in the first two rows of case 3 in table 4.

**Figure 5.**
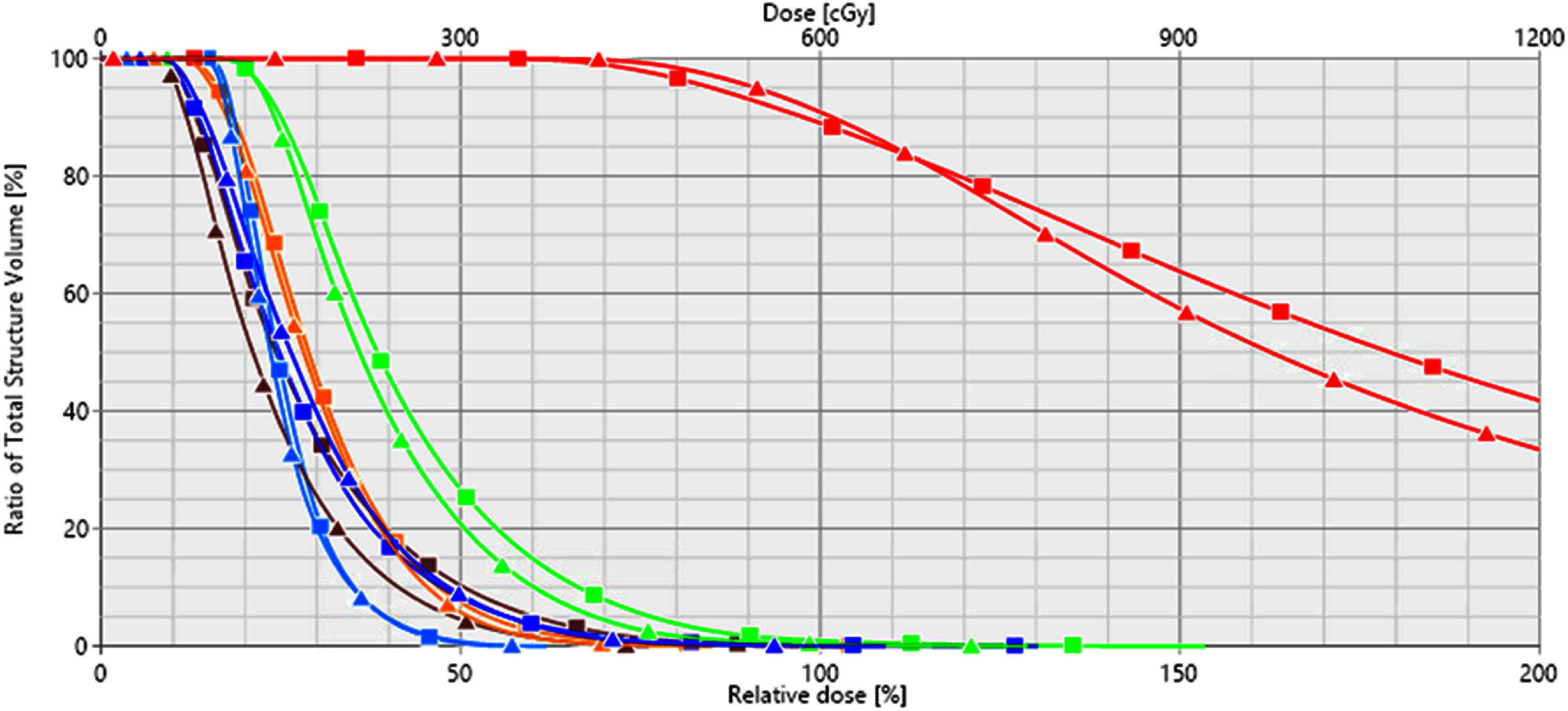
Dose-volume histogram (DVH) comparison between the original treatment plan using the actually inserted Syed applicator and the treatment plan generated for a simulated T&O applicator insertion for the fifth insertion of the case 3. DVH curves for the original Syed treatment plan are marked with squares, while curves for the simulated T&O treatment plan are marked with triangles. The red, green, orange, dark blue, light blue, and brown lines represent the DVHs of HR-CTV, bladder, sigmoid, large bowel, small bowel, and rectum, respectively.

The final model was also tested on Dataset-3, and the detailed results are shown in table 4. The model correctly selected the appropriate applicator type for all three insertions of case 1. In case 2, the model accurately suggested the appropriate type for all four insertions, including the third insertion where the treatment delivery was cancelled due to the use of a T&O applicator that resulted in unacceptable plan quality (marked as insertion index 3* in table 4). In case 3, the model correctly suggested the type for the second, third, and fourth insertions. However, for the first and fifth insertions, the suggested applicator types differed from the actually used applicators. A review of the first insertion revealed that the HR-CTV contour did not align with our conventional contouring practices (corresponding to insertion index 1(a) in table 4), containing a large inferior portion below the ovoids that was not covered by the prescription dose in the clinical plan, as shown in figure 4(a). To investigate whether this unconventional contouring contributed to the misclassification of our model, we removed the HR-CTV contour from the last five inferior CT slices (1 mm slice thickness), as shown in figure 4(b). With this modified contour that is more aligned with our institutional contouring practices, the model correctly suggested the applicator type (corresponding to insertion index 1(b) in table 4). For the fifth insertion, the model suggested that a T&O applicator would suffice. To verify this suggestion, one of our experienced medical physicists conducted a simulation study, placing the two digital ovoids of the T&O applicator on the patient’s CT images based on the central tandem location, and creating a treatment plan for the simulated T&O applicator insertion. The dose volume histograms (DVHs) of the original Syed plan and the simulated T&O plan were compared, as shown in figure 5. The comparison revealed that the T&O applicator provided a slightly higher quality plan than the Syed applicator for the fifth insertion, with slightly better *D*_90%_ dose coverage (101.2% vs. 97.5%), lower doses to OARs including bladder *D*_2cc_ (4.15 Gy vs. 4.63 Gy), sigmoid *D*_2cc_ (3.24 Gy vs. 3.44 Gy), large bowel *D*_2cc_ (3.65 Gy vs. 3.70 Gy), rectum *D*_2cc_ (3.42 Gy vs. 4.27 Gy), and comparable small bowel *D*_2cc_ dose (2.33 Gy vs. 2.32 Gy). This simulation study indicated that T&O would have been sufficient for the anatomy in the fifth insertion, with no need for the invasive Syed applicators. In case 4, while the model correctly classified the second insertion, it misclassified the first insertion as suitable for a T&O applicator, with a predicted Syed suitability score of 0.296. Similarly, a simulation study was conducted by our medical physicist and confirmed this misclassification by the model.

**Table 4.** Results obtained by our final model for the four cases of Dataset-3.

Special case	Insertion index	Actually used applicator	Ground truth applicator type	Predicted applicator type	Predicted Syed suitability score
1	1	T&O	T&O	T&O	0.079
	2	T&O	T&O	T&O	0.016
	3	Syed	Syed	Syed	0.771

2	1	T&O	T&O	T&O	0.048
	2	T&O	T&O	T&O	0.016
	3*	T&O	Syed	Syed	0.525
	4	Syed	Syed	Syed	0.701

3	1(a)	T&O	T&O	Syed	0.650
	1(b)	T&O	T&O	T&O	0.178
	2	T&O	T&O	T&O	0.039
	3	T&O	T&O	T&O	0.134
	4	T&O	T&O	T&O	0.033
	5	Syed	T&O	T&O	0.086

4	1	Syed	Syed	T&O	0.296
	2	T&O	T&O	T&O	0.033

3*: The treatment delivery of case 2’ 3rd insertion was cancelled after treatment planning due to unacceptable plan quality obtained in the resulting T&O plan;

1(a) and 1(b): correspond to model performance for case 3’ 1st insertion using original unconventional HR-CTV contour and modified contour (as shown in figure 4).

These results for Dataset-3 are also summarized in table 3, where the insertions with the ground truth applicator type as Syed are considered positive samples and those with the true type as T&O are considered as negative samples. All the ten negative samples and three positive samples were correctly classified, while one positive sample was misclassified, yielding a sensitivity of 75.0%, specificity of 100.0%, and accuracy of 92.9%. Note that the result for the first insertion of case 4 is based on the modified contour, with the original contour excluded from the evaluation of the overall model performance. By recalculating the performance metrics based on all the cases from both testing datasets, our final model achieved a sensitivity of 90.9%, specificity of 97.4%, and accuracy of 96.0%.

### 3.3. Impact of 3D distance maps on model performance

To assess the impact of incorporating 3D distance maps in the input on model performance, we repeated the experiments presented in sections 3.1 and 3.2 using only the 3D mask data as input. The comparison results of the five-fold cross-validation on Dataset-1 and the model evaluation on Dataset-2 and Dataset-3 are presented in tables 5 and 6, respectively. For ease of comparison, the results obtained using 3D distance maps, previously reported in tables 2 and 3, are also included. These results indicate that incorporating distance maps in the model input significantly improves model performance.

**Table 5.** Comparison of five-fold cross-validation results obtained by the DL models trained with and without using 3D distance maps.

Fold	With 3D distance maps		Without 3D distance maps	
	Training accuracy (%)	Validation accuracy (%)	Training accuracy (%)	Validation accuracy (%)
1	96.9	98.6	91.9	94.2
2	95.3	91.6	97.5	85.9
3	97.7	91.4	95.3	82.7
4	97.2	90.4	96.3	90.4
5	98.4	88.5	97.5	91.0

Mean ± std	97.1 ± 1.1	92.1 ± 3.8	95.7 ± 2.3	88.8 ± 4.9

**Table 6.** Comparison of model performance on Dataset-2 and Dataset-3 for the final DL models trained with and without using 3D distance maps.

	With 3D distance maps			Without 3D distance maps		
	Sensitivity (%)	Specificity (%)	Accuracy (%)	Sensitivity (%)	Specificity (%)	Accuracy (%)
Dataset-2	100.0	96.6	97.2	42.9	89.7	80.6
Dataset-3	75.0	100.0	92.9	75.0	70.0	71.4

Total	90.9	97.4	96.0	54.5	84.6	78.0

### 3.4. Computational efficiency

This study was conducted on a workstation equipped with an Intel(R) Xeon(R) Gold 6258 R CPU @ 2.70 GHz and an NVIDIA RTX A6000 GPU. The preprocessing of CTV and OAR contours to prepare model input was performed in MatLab using only the CPU, which took approximately 3 min per case (i.e. insertion). Our DL model was developed and trained using TensorFlow. The final model training using the complete Dataset-1 took approximately 20 h on the GPU. For the inference, the model’s prediction time was less than 100 ms per case, regardless of whether GPU or CPU was used.

### 3.5. Benchmark results of our implemented ML approach

The best prediction performance using the conventional ML approach was achieved with a set of eight top-ranked features, including the CTV volume size, the average lateral extension of the CTV, the average offset of the CTV, and the maximum, average, and minimum distances of the nearest 2cc bladder volume to the CTV surface, as well as the maximum and average distances of the nearest 2cc rectum volume to the CTV surface. The five-fold cross-validation results using these features and hyperparameter-tuned ML models are presented in table 7. As shown, SVM achieved an accuracy of 77.8% with a sensitivity of 71.1% and a specificity of 80.1% averaged over the five folds; RF achieved 78.7% accuracy with 69.7% sensitivity and 81.8% specificity; KNN achieved 75.5% accuracy with 79.9% sensitivity and 73.7% specificity; and DT achieved 79.1% accuracy with 65.7% sensitivity and 83.7% specificity. The soft-voting ensemble approach further improved the performance, achieving 81.3% accuracy with 79.8% sensitivity and 81.7% specificity. However, all four individual ML models and the soft-voting ensemble approach still underperformed compared to our DL model, which achieved 92.1% accuracy with 82.8% sensitivity and 95.3% specificity.

**Table 7.** Five-fold cross-validation results of four conventional ML models (e.g. SVM, RF, KNN, DT) on Dataset-1, using a set of eight top-ranked features and the optimized hyperparameters for each model. The final prediction result obtained using a soft-voting ensemble of the four models is also presented. For comparison, the corresponding five-fold cross-validation results of our proposed DL model (as reported in table 2) are also included.

Fold	SVM			RF			KNN			DT			Soft voting			Our DL method		
	Sensitivity (%)	Specificity (%)	Accuracy (%)	Sensitivity (%)	Specificity (%)	Accuracy (%)	Sensitivity (%)	Specificity (%)	Accuracy (%)	Sensitivity (%)	Specificity (%)	Accuracy (%)	Sensitivity (%)	Specificity (%)	Accuracy (%)	Sensitivity (%)	Specificity (%)	Accuracy (%)
1	73.7	88.0	84.1	68.4	84.0	79.7	89.5	76.0	79.7	63.2	94.0	85.5	84.2	90.0	88.4	100.0	98.0	98.6
2	71.4	68.0	69.0	81.0	74.0	76.1	76.2	66.0	69.0	85.7	68.0	73.2	81.0	68.0	71.8	90.5	92.0	91.6
3	68.2	90.2	84.3	72.7	86.9	83.1	86.4	85.2	85.5	63.6	88.5	81.9	86.4	90.2	89.2	90.5	91.7	91.4
4	78.9	73.1	74.6	63.2	82.7	77.5	84.2	63.5	69.0	63.2	86.5	80.3	84.2	80.8	81.7	70.0	98.1	90.4
5	63.2	81.4	76.9	63.2	81.4	76.9	63.2	78.0	74.4	52.6	81.4	74.4	63.2	79.7	75.6	63.2	96.6	88.5

Avg	71.1	80.1	77.8	69.7	81.8	78.7	79.9	73.7	75.5	65.7	83.7	79.1	79.8	81.7	81.3	82.8	95.3	92.1

Std	5.3	8.5	5.8	6.7	4.3	2.5	9.4	8.0	6.4	10.8	8.8	4.6	8.5	8.2	6.9	15.5	3.2	3.8

The voting results of the four final ML models, retrained on the complete Dataset-1, for Dataset-2 and Dataset-3 are summarized in table 8. The soft-voting ensemble yielded a sensitivity, specificity and accuracy of 71.4%, 93.1%, and 88.9% on Dataset-2, and 75.0%, 90.0%, and 85.7% on Dataset-3. When combing results across both testing datasets, the soft-voting approach achieved an overall sensitivity of 72.7%, specificity of 92.3%, and accuracy of 88.0%. In comparison, our DL model achieved a better performance with a sensitivity of 90.9%, specificity of 97.4%, and accuracy of 96.0%.

**Table 8.** Voting results of the four final ML models for Dataset-2 and Dataset-3. The corresponding results of our final DL model are also presented for comparison.

	Soft voting			Our DL model		
	Sensitivity (%)	Specificity (%)	Accuracy (%)	Sensitivity (%)	Specificity (%)	Accuracy (%)
Dataset-2	71.4	93.1	88.9	100.0	96.6	97.2
Dataset-3	75.0	90.0	85.7	75.0	100.0	92.9

Total	72.7	92.3	88.0	90.9	97.4	96.0

Overall, these benchmark results demonstrate that while conventional ML models with carefully engineered handcrafted features provided decent predictive performance, our DL model consistently outperformed the ML models across all evaluation metrics.

## 4. Discussion

Data scarcity is often a limiting factor for DL applications in medical settings, posing a significant challenge to achieving generalizability of DL models across large and diverse patient populations. This limitation is particularly relevant to our study, with only 184 patient cases available from the HDR brachytherapy program at our institution. To maximize the utility of this limited data, we used five-fold cross-validation on 163 cases collected in our initial study phase (Dataset-1) to optimize hyperparameters. During cross-validation, a notable variation in validation accuracy across folds (ranging from 88.5% to 98.6%) was observed. This variation is likely due to our limited dataset size, which made it challenging to achieve balanced data splits and increased sensitivity to outliers or atypical data in certain folds. Averaging performance across folds reduced the impact of any single fold that contains outliers or atypical data, providing a more reliable model evaluation. After optimizing hyperparameters, we trained the final model on the full Dataset-1 and evaluated it on two independent testing sets: 17 cases treated after the initial data collection (Dataset-2) and 4 cases with complex clinical scenarios (Dataset-3). Among these testing cases, one Syed insertion and one T&O insertion were misclassified by our model. Our future work will involve collecting more cases to further validate our method and incorporating misclassified cases into the training data for continued improvement.

The geometry of the selected applicator determines the radioactive source pathway within the patient’s body, making appropriate applicator selection essential for achieving optimal dose distribution. In case 2 of Dataset-3, the mis-selection of a T&O applicator for the third insertion resulted in a clinically unacceptable plan, with both bladder and rectum *D*_2cc_ doses exceeding clinical tolerances. Consequently, the treatment was canceled, and the patient underwent a fourth insertion with a Syed applicator, which significantly improved dosimetry. Specifically, the Syed plan maintained equivalent target coverage (HR-CTV *D*_90_ = 100% of the prescription dose, 5.23 Gy) while reducing hot spots (V200%: 19% vs. 28.7%) and lowering *D*_2cc_ doses for the bladder (3.73 Gy vs. 5.29 Gy), rectum (2.17 Gy vs. 4.87 Gy), small bowel (2.15 Gy vs. 2.96 Gy), and large bowel (1.04 Gy vs 1.30 Gy). Although the sigmoid *D*_2cc_ dose increased slightly (3.40 Gy vs. 2.91 Gy), it remained within clinical tolerance. Our model correctly identified that a Syed applicator should have been used for the third insertion, which would have prevented treatment cancellation and delay, highlighting its clinical relevance. It is also important to note that the clinical relevance of applicator selection extends beyond its dosimetric impact, as our study not only aims to enable optimal dose distribution, but also seeks to minimize the unnecessary use of invasive Syed applicators to reduce patient discomfort, associated risks, and resource demands. For instance, in case 3, our simulation study for the fifth insertion supported our model’s recommendation that a T&O applicator would have been sufficient, thereby avoiding an unnecessary invasive insertion of Syed applicator.

We would like to mention that during our five-fold cross-validation, we evaluated different classification thresholds and identified the optimal value of 0.494 based on the average validation accuracy across folds (92.2 ± 3.8%). However, given the limited sample size of our training dataset, this threshold selection might be biased by its data distribution. Therefore, we decided to use the default threshold of 0.5 in our experiments, which closely aligns with the identified optimal threshold of 0.494, and the choice between 0.5 and 0.494 did not change the classification results of the two independent testing datasets. The primary goal of our study is to demonstrate the feasibility of our proposed method. In future work, as we collect a more diverse and comprehensive dataset, we will refine the threshold selection process. Additionally, while our current experiments focused on classification accuracy, that is, optimizing hyperparameters during five-fold cross-validation to maximize accuracy, different clinical priorities may warrant threshold adjustments. For instance, if minimizing the risk of treatment cancellations due to unacceptable plan quality from T&O applicators is the priority, a threshold favoring higher sensitivity with clinically acceptable specificity may be more appropriate. Conversely, if reducing unnecessary use of the invasive Syed applicator to minimize the associated procedural risks is preferred, a threshold emphasizing higher specificity with acceptable sensitivity may be more suitable. Ultimately, this decision would depend on physician’s preferences and their assessment of the clinical consequences of each scenario.

Our study currently focuses on the selection between Syed and T&O applicators due to the limited availability of patient data. Although triple tandems and multi-channel interstitial cylinders are occasionally used as alternatives to Syed applicators at our institution, the small sample size of patients treated with these applicators makes it infeasible to train a comprehensive model that includes all applicator types at this stage. We are actively collecting new patient data, and plan to expand our proposed method to incorporate additional applicator types as the dataset grows. While our benchmark results using four conventional ML models and a soft-voting ensemble demonstrated that our DL model consistently outperformed these ML approaches, a key and broader advantage of DL over traditional ML is its ability to automatically learn relevant features from the data, eliminating the need for manual feature engineering. This inherent strength of DL is central to our study, as our model directly takes the 3D mask and distance data that represent the geometries of the CTV and OARs as input. While incorporating additional applicator types would require expanding the training dataset, the DL model is inherently designed to adapt to new applicators without the need for manual identification or engineering of new relevant features specific to those applicators.

We would like to mention that our HDR brachytherapy program has recently begun using T&O applicators in combination with a few free-hand interstitial needles in select cases or subsequent insertions to improve dose distribution. As a result, 19 of the 29 T&O insertions in Dataset-2 included 2–4 additional free-hand needles. Since these additional needles were few in number and did not significantly alter the overall dose distribution pattern of T&O applicators, most of these hybrid insertions were still classified as T&O suitable cases by our model. However, one insertion involving three extra needles was misclassified as Syed. While it would be desired for our model to also predict when extra interstitial needles are needed to go with T&O applicators, the limited number of hybrid cases makes training for this classification impractical. A potential solution could involve developing a DL model for 3D dose prediction of T&O plans to assess whether free-hand needles are necessary (Ma *et al* 2021, Kallis *et al* 2023, Li *et al* 2023). However, there are two key challenges that need to be addressed to ensure the accuracy of the dose prediction. First, the dose distribution of a T&O plan largely depends on the selected applicator’s geometry (e.g. central tandem angle of 15, 30 or 45° and ovoid sizes of mini, small, medium, or large for the left and right ovoids based on patient anatomy). The limited number of patient cases for each specific applicator geometry poses a challenge for robust network training. Second, the dose distribution of a T&O plan is also affected by the physician specified trade-offs between target coverage and OAR sparing, which vary based on the patient’s prior EBRT dose. This variation in trade-offs across the collected T&O plans presents another challenge for dose prediction. To address these issues, in our future work we will generate simulated plans for different T&O applicator geometries with simulated insertion locations, as well as a range of plans reflecting diverse trade-offs. This will provide sufficient data to train a DL model capable of accurately predicting 3D dose distributions tailored to both applicator geometry and physician preferred trade-off.

In addition to data limitation, the performance of our model is also constrained by data quality. For instance, the results with the two sets of HR-CTV contours (i.e. the original and modified contours shown in figure 4) for the first insertion of case 3, as shown in table 4, demonstrate that the model’s accuracy relies on consistent contouring practices and is sensitive to contouring variability. This variability could pose challenges when applying our model to data from other institutions or expanding the training dataset to include cases from multiple institutions. Moreover, contouring for HDR brachytherapy typically needs to be completed within a limited timeframe, which can result in suboptimal contouring quality and hence impact the model’s training and performance. Advances in auto-contouring present a promising solution to alleviate the time constraint and enhance contour consistency (Duprez *et al* 2023, Mohammadi *et al* 2021, Wang *et al* 2023). By providing an initial contour for physicians to review and refine, auto-contouring tools may reduce manual effort and help standardize delineations. Standardizing contouring protocols across institutions would be an ideal approach to further reduce variability, however this will require time and collaborative efforts. As an interim solution, we could introduce synthetic variations in contours, such as contour smoothing or minor modifications, as data augmentation during training to enhance model resilience to slight contouring differences, while these synthetic contours should be verified to ensure that such variations do not alter the optimal applicator type. Another approach might involve transfer learning (Kamath *et al* 2019), using a smaller dataset from a new institution to fine tune the model and adapt it to the institutional-specific contouring convention. Alternatively, an ensemble approach (Ganaie *et al* 2022), training separate models on datasets from different institutions and using averaging, voting, or stacking techniques, may help reduce sensitivity to minor contour inconsistencies and improve robustness in multi-institutional settings.

Another challenge regarding data quality stems from the original applicator selections in our dataset, which were made based solely on the physician’s experience and judgement and may not always reflect the optimal choice, particularly in atypical cases, potentially affecting data consistency. To enhance the quality of our training data, as detailed in section 2.1, an experienced medical physicist reviewed the applicator type in each case of our dataset to determine the ground truth label, independently of the original applicator choice. However, this manual review process, particularly for cases with Syed applicators, is labor-intensive and may be challenging to scale when expanding data collection across multiple institutions in future. As a practical solution, our current model could serve as a quality assurance tool for incoming new data, flagging cases where the model’s applicator recommendation differs from the physician’s choice for a further manual review.

In this retrospective study, we collected contour data from post-insertion treatment planning, as not all patients had pre-insertion MRI scans before their HDR brachytherapy course at our institution. At our institution, pre-insertion MRI scans are typically ordered for Syed applicator candidates to aid in preplanning needle placements to guide subsequent insertion procedure; T&O applicator candidates usually do not receive pre-insertion MRI scans; instead, a post-insertion MRI is acquired on the day of the procedure. Given this constraint, our model was trained on post-insertion anatomy in this study. We plan to integrate our model, trained on post-insertion anatomy, into our clinical workflow as a post-insertion quality assurance tool with IRB approval, to provide feedback to physicians on their applicator choices. Over time, this feedback loop could help physicians evaluate and refine their selection processes. We also fully recognize the clinical relevance of selecting the appropriate applicator type before the insertion procedure. As a next step, we plan to conduct a prospective study with IRB approval, acquiring pre-insertion diagnostic MRI scans for every new LACC patient and applying our model to predict the appropriate applicator type. Model predictions will be compared with physician selections and validated through dosimetric plan quality assessments. It is worth noting that in our current retrospective study, we used tandem location on the post-insertion CT images as an indicator of the cervical canal. Because the cervical canal can be distinguished and delineated on pre-insertion T2-weighted MRI, the absence of tandem on the pre-insertion MRI should not hinder the model’s applicability in the pre-insertion setting. However, applicator insertion can induce significant anatomical changes, such as alterations in uterus orientation and shifts in tumor position relative to nearby OARs, which may alter the indication for the appropriate applicator type. Tambas *et al* (2021) reported this issue in a prospective study of 24 LACC patients. Among 15 patients initially indicated for interstitial brachytherapy based on pre-insertion MRI, the indication was no longer present post-insertion in 5 patients. Conversely, 9 patients initially indicated for intracavitary brachytherapy presented indications for interstitial brachytherapy after insertion. To address this challenge in pre-insertion prediction, one potential solution is to develop a separate DL model trained on paired pre- and post-insertion images to predict post-insertion anatomy from pre-insertion anatomy (Ghosh *et al* 2022), enabling our model to work in the pre-insertion setting while accounting for potential anatomical changes induced by applicator placement. Alternatively, or ultimately, we envision an intraoperative workflow that includes MR imaging after central tandem placement, which is the main source of anatomical changes. A rapid prediction of appropriate applicator type could then be made based on this intermediate anatomy, supported by DL -based auto-contouring and followed by automated preplanning if interstitial brachytherapy is suggested.

The black-box nature of DL models may present challenges in gaining clinical trust during clinical implementation. To address this, a key focus of our future work is to develop explainable AI models (Saraswat *et al* 2022, Dwivedi *et al* 2023). Additionally, our group is also working on related projects, including 3D dose prediction for T&O cases, and deep reinforcement learning-based automatic preplanning for patients identified to receive Syed applicators. These initiatives aim to fully integrate AI-driven decision support into clinical practice to enhance the treatment quality of HDR brachytherapy.

While our model is designed to assist less experienced physicians in selecting the appropriate applicator type, which is a critical step in treatment planning and a prerequisite for optimal plan quality, successful insertion remains highly dependent on the physician’s skill, experience, and the patient’s anatomy. Our study focuses specifically on supporting applicator selection. Meanwhile, significant efforts have been made to improve insertion accuracy, including virtual reality training to enhance insertion skills (Taunk *et al* 2021, Al Balushi *et al* 2022) and robotic-assisted techniques to aid the insertion (Rabatic *et al* 2015a, 2015b). We believe that combining our model with these advancements will collectively refine procedural quality in HDR brachytherapy, ultimately improving treatment outcomes for LACC patients.

## 5. Conclusions

In this study, we developed a DL-based decision-support tool for applicator selection in LACC HDR brachytherapy, focused on choosing between Syed and T&O applicators. Our model achieved an average validation accuracy of 92.1 ± 3.8% in five-fold cross-validation with a total of 372 applicator insertions from 163 patient cases. When tested on two independent datasets including 50 applicator insertions from 21 new patients, our final model yielded an overall accuracy of 96.0%, with a sensitivity of 90.9% and a specificity of 97.4%. For benchmarking, we also employed four conventional ML models combined with a soft-voting ensemble. The best performance from this ML approach yielded an average accuracy of 81.3 ± 6.9% in the five-fold cross-validation and an overall accuracy of 88.0% with 72.7% sensitivity and 92.3% specificity on the two independent testing datasets. The benchmark results demonstrate that our DL model outperforms these conventional ML approaches in this applicator selection task. The promising performance, particularly with the four complex cases, demonstrates the potential of our DL model as an effective quality assurance tool, providing feedback to physicians on their applicator choices, ultimately supporting continuous improvement in clinical decision-making. Our future work will focus on expanding the training dataset to further validate and enhance the model’s performance and robustness, as well as extending its application for prospective applicator selection. Improving the applicator selection process is expected to yield better dose distributions and potentially improve treatment outcomes.

## Data Availability

The data cannot be made publicly available upon publication due to legal restrictions preventing unrestricted public distribution. The data that support the findings of this study are available upon reasonable request from the authors.
